# Tele-Group Cognitive Behavioural Family Intervention for Schizophrenia-Spectrum Disorders and Their Caregivers: A Feasibility Randomised Controlled Trial

**DOI:** 10.3390/healthcare14142231

**Published:** 2026-07-22

**Authors:** Dennis Chak Fai Ma, Cheuk Kin Tang, Cheyenne I Ying Chan, Grace Wing Ka Ho, Sau Fong Leung, Flora Ki Nga Wong, Fan Ngan, Lok Tung Yeung, Daniel Bressington, Sherry Kit Wa Chan

**Affiliations:** 1 School of Nursing, The Hong Kong Polytechnic University, Hung Hom, Hong Kong SAR, China; dennis.cf.ma@polyu.edu.hk (D.C.F.M.); cheyenne.chan@polyu.edu.hk (C.I.Y.C.); grace.wk.ho@polyu.edu.hk (G.W.K.H.); sau.fong.leung@polyu.edu.hk (S.F.L.); wknflora@gmail.com (F.K.N.W.); fannynganfan326@gmail.com (F.N.); anitayeung2016@gmail.com (L.T.Y.); daniel.bressington@cmu.ac.th (D.B.); 2 Department of Psychiatry, Kowloon Hospital, Kowloon City, Hong Kong SAR, China; tangck1@ha.org.hk; 3 Faculty of Nursing, Chiang Mai University, Chiang Mai 50200, Thailand; 4 Department of Psychiatry, School of Clinical Medicine, Li Ka Shing Faculty of Medicine, The University of Hong Kong, Pokfulam, Hong Kong SAR, China; 5 Department of Psychiatry, Queen Mary Hospital, Pokfulam, Hong Kong SAR, China

**Keywords:** cognitive behavioural intervention, family intervention, telemental health, schizophrenia, caregivers

## Abstract

**Background:** Family-based interventions are effective in mitigating the risk for relapse of schizophrenia. However, the accessibility of these interventions is scarce in many clinical settings. A group-based brief cognitive behavioural intervention facilitated by a therapist using videoconferencing may help address this practice gap and improve the high treatment disengagement that occurs in interventions delivered in self-paced web-based forums or mobile applications. **Objective:** To examine the feasibility, acceptability, and safety of an online group-based cognitive behavioural family intervention for dyads of individuals with schizophrenia-spectrum disorders and caregivers. **Methods:** This feasibility study adopted a parallel-group, assessor-blind randomised controlled trial with a twelve-week post-intervention follow-up as well as individual semi-structured interviews. Participants were randomly assigned to the intervention group [i.e., tele-group cognitive behavioural family intervention (tgCBFI) group] and the treatment-as-usual group. Both groups also received biweekly brief telephone support. The feasibility and acceptability were assessed by the recruitment rate, intervention completion rate, retention rate and participants’ service satisfaction. Safety was measured by the number of adverse events. **Results:** Most intervention group participants (85.7%) attended all six online group sessions (six service user-caregiver dyads, corresponding to 12 participants), while 100% of participants attended the per-protocol number of sessions (≥four sessions). The study recruitment rate was 16.4%, while the study retention rate for follow-up assessments was 95.8%. No adverse events were reported throughout the study. Five themes were generated to illustrate the benefits of and recommendations for the tgCBFI programme to complement the quantitative findings. **Conclusions:** The preliminary results suggested that the use of videoconferencing to deliver group-based cognitive behavioural intervention was partially feasible and provided exploratory estimates suggesting possible improvement in psychiatric symptoms for individuals with schizophrenia-spectrum disorders, warranting a fully powered trial. Trial Registration: prospectively registered at ClinicalTrials.gov NCT05808244.

## 1. Introduction

Schizophrenia-spectrum disorders affect about 0.33% of the world population [1]. The lifetime prevalence of schizophrenia-spectrum disorders in Hong Kong was estimated to be 2.17% [2], and the long-term recovery rate was about 25% even in the presence of an early intervention for psychosis programme [3,4]. The number of individuals with schizophrenia-spectrum disorders receiving public mental health services in 2021 was 50,400 [5], approximately 0.68% of the Hong Kong population. The positive symptoms (e.g., auditory hallucinations and persecutory delusions) and negative symptoms (e.g., social withdrawal and avolition) cause substantial suffering to people with schizophrenia and their families, both functionally and financially. Poor family communication and support have been shown to preclude individuals with schizophrenia from reintegrating into the community and to increase their family caregivers’ psychological distress and perceived care burden [6]. Expressed emotion refers to the quality of family atmosphere, interactions and functioning, comprising the following three elements: emotional overinvolvement, hostility, and critical comments [7]. A recent meta-analysis revealed that family interventions were superior to other psychosocial interventions in relapse prevention [8], and familial expressed emotion, particularly critical comments, was a robust predictor of relapse in schizophrenia [9].

The current mainstream approaches of family intervention for schizophrenia include supporting family caregivers to reduce perceived care burden and caring stress [10] and working with the whole family to reduce the symptom severity of patients [11]. Rooted from these two approaches of behavioural and structural family interventions [12], cognitive behavioural family intervention (CBFI) was developed to focus on cognitive appraisals in family relationships and interpersonal events between service users and their families [13]. The involvement of both service users and family caregivers in psychotherapeutic sessions can facilitate their communication and mutual understanding in certain interpersonal, familial, and mental health issues [13]. A meta-analysis indicates that CBFI is superior to treatment as usual in improving positive and negative symptoms of individuals with severe mental illnesses immediately [14]. Therefore, it could be an effective patient-centred, family-involved psychosocial intervention with less time and resource restraint compared with full-scale family therapy, applying the key concepts and skills in cognitive behavioural therapy (CBT) in the family context. It aims to modify the thoughts and beliefs or cognitive appraisal of interpersonal events among service users and families through group process with the help of CBT skills to improve the family communication and dynamics.

Innovative intervention programmes using digital technology, such as self-paced web-based psychoeducation and automated smartphone applications, to improve accessibility to mental health information and care have been widely implemented in mental health services [15]. A recent systematic review supported that telepsychiatry is feasible and acceptable for individuals with severe mental illness and their family caregivers [16]. However, without interactive, personalised, and authentic human support, disengagement rates from these programmes are usually high [17]. Particularly, another two recent reviews support that the use of group videoconferencing with protocol-based workbook may be one of the future digital mental health trends to complement the face-to-face therapeutic consultations and treatments for individuals with schizophrenia-spectrum disorders [18,19]. The development of this tele-group cognitive behavioural family intervention programme is to offer optimal and effective psychiatric care in balance for the desirable treatment engagement and leveraging the advantages of telemental health, such as increased flexibility and accessibility. This study adopted quantitative and qualitative methods with the aim of examining the feasibility and acceptability of delivering the tgCBFI programme to dyads of individuals with schizophrenia and their family caregivers and generate preliminary evidence on the effectiveness of tgCBFI on outcomes of both service users and their family caregivers.

## 2. Methods

### 2.1. Study Design

This was a parallel-group, assessor-blind, feasibility randomised controlled trial with a twelve-week post-intervention follow-up. Individual semi-structured interviews for service users and caregivers were also conducted to collect feedback to complement the quantitative data. This study was prospectively registered at ClinicalTrials.gov (NCT05808244). All procedures involving human subjects/patients were approved by The Hong Kong Polytechnic University Institutional Review Board (HSEARS20221011011-1), the Hospital Authority Kowloon Central/Kowloon East Cluster Research Ethics Committee (KC/KE-22-0170/FR-1), and The University of Hong Kong/Hospital Authority Hong Kong West Cluster Institutional Review Board (UW22-173). This study commenced in April 2023, and the first recruited service user-caregiver dyad was on 30 Jun 2023. The last data follow-up was in September 2024, and the data analysis was completed in November 2024. This study was reported in line with the CONSORT 2010 statement extension to randomised pilot and feasibility trials [20], and no amendments were made after the trial commenced.

### 2.2. Participant Recruitment and Randomisation

This study was in collaboration with the community psychiatric unit at a general hospital of the Hospital Authority in Hong Kong. The participants were recruited from the outpatient unit by convenience sampling and screened by a senior nurse. In each cohort of participant recruitment, a briefing session was arranged for all participants to explain the study logistics, consent form procedure, and the use of Zoom videoconferencing by an independent and trained research assistant (RA), who was a registered psychiatric nurse with five years of clinical experience and a master’s degree. Before baseline assessment, a random sequence number list and randomisation using the Sealed Envelope website (https://www.sealedenvelope.com) were generated by the independent RA, who had no knowledge of the participants. The dyads were randomly assigned to either the intervention group or the treatment as usual group using 1:1 block randomisation (size of 4, 6, or 8). The permuted block size aimed to maintain less predictability of allocation and balance the group size. Once the group assignment and baseline assessments were done, the participants allocated to the intervention group were informed to attend the first session accordingly. Allocation was concealed from the outcome assessor, who was blinded to group allocation at the baseline and follow-up assessments. Participants were reminded not to disclose their group assignment or intervention experience during the Zoom-based assessments.

### 2.3. Inclusion and Exclusion Criteria for Participants

The inclusion criteria for service users were (i) current diagnosis of schizophrenia, schizotypal, and delusional disorders (F20–F29), based on ICD-10 made by the treating clinicians, (ii) aged 18–64, and (iii) able to read, write and communicate in Cantonese to provide written consent. While ICD-10 is routinely used for diagnostic coding and clinical documentation in Hong Kong’s public hospitals, DSM-5 is commonly referenced for international research comparability. Schizophrenia, schizotypal, and delusional disorders (F20–F29) in ICD-10 diagnoses would broadly correspond to DSM-5 schizophrenia spectrum and other psychotic disorders. Diagnoses were determined by treating clinicians based on clinical assessment and medical records. The present study relied on ICD-10 diagnoses made in routine clinical practice and did not independently re-diagnose participants according to DSM-5 criteria. The exclusion criteria were (i) having a co-morbidity of learning disability, organic/neurological conditions, or substance misuse disorder, and (ii) living in a hostel. The inclusion criteria for family caregivers were (i) aged above 18, (ii) able to communicate in Cantonese, (iii) live with service users, and (iv) nominated by the service users. The exclusion criterion was having active psychiatric conditions.

### 2.4. Tele-Group Cognitive Behavioural Family Intervention (tgCBFI) Group

It was a six-week online group intervention programme for dyads of service users and caregivers (i.e., one service user and one family caregiver) using the CBFI framework. The group intervention included a maximum of eight dyads of participants at a time. It aimed to enable service users and caregivers to understand each other’s perspectives, fostering better family relationships by understanding their cognitive model of CBT. Based on the stress-vulnerability family coping model [21] and integrated socio-developmental cognitive model [22], the therapeutic mechanism of CBFI was to reduce the familial expressed emotion or familial stress/dysfunction to foster better family communication and functioning within a family that cultivates and sustains a positive family climate, enabling family members to live in harmony and supporting each other.

It comprised six sessions, and each of the sessions lasted for a maximum of one and a half hours. There were four main themes in the programme. First, the assessment of family background and exploration of one resolved conflict issue between caregivers and service users were explored. Feedback given by other participants and discussion over this issue were encouraged to foster family mutual support. Second, signs and symptoms of schizophrenia-spectrum disorders and caring stress of carers were discussed, followed by educating the cognitive model of CBT and introducing family models on schizophrenia-spectrum disorders to provide basic knowledge information and psychological support. Third, practising basic CBT techniques such as using the dysfunctional thought record worksheet among the participants and coping skills for the family based on the identified conflicts were stressed to allow a professional-guided platform of doing perspective-taking and pacifying family relationships. Finally, consolidation of the previous sessions and relapse prevention measures was addressed. This intervention programme was developed and delivered by the first author, who was an early-career researcher, a qualified cognitive therapist, and a medication management facilitator with ten years of clinical experience in adult psychiatric nursing. The treatment manual and booklet were reviewed and commented on by the research team, including experienced psychiatric nurses, a clinical psychologist and a psychiatrist. All groups followed the same manualised content, session sequence, duration, and online delivery format. No planned differences in content or delivery occurred between cohorts. Intervention standardisation was supported through the use of the structured treatment manual, standardised session slides, and participant booklet. Table 1 is provided to illustrate the treatment content. Although psychotherapeutic components are the main course of this intervention, the unsophisticated introduction to psychiatric medications, apart from basic signs and symptoms of schizophrenia-spectrum disorders shared in sessions two and three, would unavoidably be touched on in the psychoeducation, open discussion and question-and-answer sessions, as the participants would expect to learn and clarify the basics of the medications from healthcare professionals. Individualised medication titration, augmentation, switching, or tapering would not be suggested, and seeking consultations with treating clinicians was highly advised, particularly during scheduled community psychiatric case management outreach and psychiatric outpatient appointments. The full tgCBFI intervention materials are available from the first author upon reasonable request.

Participants in the tgCBFI group would continue to receive treatment as usual (TAU) care, as well as bi-weekly ten-minute telephone support in order to keep participants engaged throughout the study period. The calls did not include any structured psychotherapy using CBT techniques or skills in family intervention. The independent RA would briefly monitor their physical and mental state using a table form and remind them to attend the next session.

### 2.5. Treatment as Usual (TAU) Group

Participants randomised into this group continued receiving TAU as indicated, including the integrated community psychiatric care with psychiatrist outpatient follow-up and the community psychiatric team, which consists of multidisciplinary professionals providing regular home visits and continuous community support. In the hope of keeping participants engaged in the study, bi-weekly ten-minute telephone support was also offered to participants in this group.

### 2.6. Safety Monitoring

Safety was monitored throughout the study implementation period. During the briefing session, all participants received a rules and regulations sheet for attending this online intervention group, such as respectful manners, attending from home and no recordings. During the study implementation, participants joined the Zoom sessions via a protected link with a passcode and a waiting room. The facilitator ensured all participants turned on the camera at the start of every session and reminded them of group confidentiality, particularly in the first session. Participants were encouraged to report any distress, symptom deterioration, family conflict, privacy concerns, or other adverse experiences to the research team during the sessions or telephone support. If a participant showed marked distress or disclosed risk during the study implementation, the facilitator or RA would pause the discussion where appropriate, contact the participant individually, notify the research team, and refer the participant to the treating clinical team or emergency services according to risk level.

### 2.7. Outcome Assessments

The primary outcomes of this feasibility study were feasibility, acceptability, and safety. These outcomes were used to determine whether the online group intervention could be readily scaled up to a definitive randomised controlled trial. The secondary outcomes included psychiatric symptom severity and perceived expressed emotion among service users, caregiver burden, and caregivers’ anxiety and depressive symptoms. Qualitative feedback was also collected from participants in the tgCBFI group through individual semi-structured interviews.

### 2.8. Feasibility, Acceptability, and Safety

Feasibility was assessed using the recruitment rate and intervention completion rate. Acceptability was assessed using the study retention rate and service satisfaction. The satisfaction was assessed by the 8-item Client Satisfaction Questionnaire (CSQ-8). CSQ-8 measures the satisfaction level of participants after completing a health service [23]. It is an interviewer-rated questionnaire that consists of eight items using a four-point Likert scale. The total score ranges from 8 to 32. The internal consistency was 0.91, and it was negatively associated with the dropout rate (*r* = −0.37, *p* < 0.01). A higher score indicates a higher level of service satisfaction. Safety was assessed using reported adverse events or adverse effects during the study period. Adverse events were defined as any undesirable medical, psychological, or social event occurring during the study period. Serious adverse events included psychiatric hospitalisation, suicide attempt, severe symptom exacerbation requiring urgent clinical intervention, violent incident, or other events requiring emergency care. Safety was monitored through facilitator observation during sessions, participant and caregiver self-report, and follow-up assessments. Any safety concerns were discussed by the research team and referred to the treating clinical team, where appropriate.

### 2.9. Progression Criteria for Future Definitive Clinical Trials

Progression criteria to inform amendments and improve future clinical trials were retrospectively formulated. A traffic light system was adapted to illustrate the criteria for recruitment, intervention protocol adherence, and outcome data completion [24]. However, there was no local pragmatic and internal data to inform the operational cut-offs for these criteria figures specific to psychiatric settings, considering the challenges in funding capacity and university–industry collaboration endeavour. Based on two international guidance papers on progression criteria [24,25], we operationally proposed the criteria for red, amber, and green lights for recruitment (<50%, 50–<80%, ≥80%), intervention protocol adherence (<40%, 40–<80%, ≥80%), and outcome data completion (<40%, 40–<80%, ≥80%). The suggested corresponding follow-up strategies for red, amber, and green lights were “substantial review, amendment, and consideration for stopping scalability”, “moderate review and amendment with exploration of the possibility of scalability”, and “minor review and amendment with continued scalability”, respectively.

### 2.10. Positive and Negative Syndrome Scale (PANSS) for Service Users

It measures the positive and negative symptoms of people with psychosis [26]. It is an interviewer-rated questionnaire with thirty items using a seven-point Likert scale with three subscales. The subscale scores of positive and negative symptoms range from 7 to 49, while those of general psychopathology ranges from 16 to 112. A higher score indicates a higher level of severity of psychiatric symptoms. An independent RA, who was an advanced practice nurse with five years of clinical experience and a master’s degree, was trained to use the interview schedule to conduct the PANSS assessment.

### 2.11. Concise Chinese Level of Expressed Emotion Scale (CCLEES) for Service Users

It measures the level of expressed emotion of family caregivers experienced by the service user [27]. It is a self-rated questionnaire with twelve items using a four-point Likert scale with three subscales to be completed by the service users. The total score ranges from 12 to 48. The internal consistency varied from 0.75 to 0.77, and it was positively associated with a gold standard Camberwell Family Interview (CFI) (ρ = 0.8, *p* = 0.003). The cutoff scores of high expressed emotion are either ≥13 in the subscales of criticism and hostility or ≥15 in overemotional involvement. A higher score indicates a higher level of expressed emotion.

### 2.12. Family Burden Interview Schedule (FBIS) for Caregivers

It measures the perceived care burden of family caregivers [28]. It is a self-rated questionnaire with 25 items using a three-point Likert scale with six subscales. The total score ranges from 0 to 50. The internal consistency was 0.87, and a higher score indicates a higher level of perceived family care burden.

### 2.13. Hospital Anxiety and Depression Scale (HADS) for Caregivers

It measures the levels of anxiety and depression of family caregivers [29]. It is a self-rated questionnaire with fourteen items using a four-point Likert scale with two subscales. The subscale scores of anxiety and depression range from 0 to 21. The internal consistency varied from 0.77 to 0.82, and the criterion validity was supported by identifying depression and anxiety cases. A higher score indicates a higher level of mood disturbance.

All the abovementioned self-report Chinese questionnaires (e.g., CCLEES, FBIS, and HADS) were delivered online to participants using Qualtrics, while Zoom interviews were conducted in Cantonese by an independent and trained RA, who was an advanced practice nurse with five years of clinical experience and a master’s degree, to assess the service satisfaction (CSQ-8) of service users and caregivers and positive and negative symptoms (PANSSs) of service users.

### 2.14. Individual Semi-Structured Interviews for Service Users and Caregivers

All participants in the tgCBFI group were invited to participate in these face-to-face individual interviews after the study to express their opinions and feedback on the research project, with the aim of exploring participant experiences and acceptability of the intervention. The interviews were conducted in a meeting room of a university by an independent and trained RA, who was an advanced practice nurse with five years of clinical experience and a master’s degree, and was not involved in participant recruitment, randomisation, and intervention delivery. An interview guide, consisting of thirteen questions categorised into five topics, including general feedback and recommendations, was used to facilitate the interviews. The interviews were conducted in Cantonese, audio-recorded with consent and lasted up to forty-five minutes.

### 2.15. Sample Size Estimation for the Feasibility Randomised Controlled Trial

By rule of thumb, twelve dyads per group were suggested for this feasibility RCT [30]. Taking account of a twenty percent attrition rate and two parallel groups, the required sample size at baseline would be thirty dyads of service users and their family caregivers (fifteen dyads in each group).

### 2.16. Data Analysis

Descriptive statistics were used to summarise the demographic characteristics and baseline measures, where continuous variables are presented as means and standard deviations, and categorical variables are presented as frequencies and percentages. Given the small sample size and the feasibility design of this study, baseline characteristics were primarily interpreted descriptively. Clinical outcome analyses were exploratory and hypothesis-generating. Change scores (e.g., post-treatment data minus baseline data) were used to describe change from baseline and compare exploratory between-group differences. The Mann–Whitney test was used to analyse the between-group treatment effect. The treatment effect size was also computed and expressed in Hedges’ *g* to indicate the magnitude of the exploratory treatment effect. Data were analysed according to the intention-to-treat (ITT) principle, whereby all participants were included in the analysis once randomised. For participants who withdrew or had missing follow-up data, the Last Observation Carried Forward (LOCF) method was used to impute missing outcome values. LOCF was used because there was minimal missing outcome data (one service user). Although outcome variability is reduced by assuming no change for one participant, the bias is likely to be small, and the efficacy analysis was exploratory in nature in this feasibility study. Furthermore, a complete case sensitivity analysis was conducted to assess the consistency of the findings between the two analytical approaches. The significance level was set as 0.05. R version 4.4.2 was used to analyse the collected data.

Thematic analysis was used to treat and manage the qualitative data. The transcription, coding and clustering of the themes (i.e., themes and subthemes) were performed by an independent and trained RA under the supervision of the first author, based on the six-phase approach and techniques described in the literature [31]. Coding was primarily inductive, while being informed by the feasibility aims of exploring acceptability, perceived benefits, barriers, and recommendations for refinement. One trained RA conducted initial transcription, followed by data coding after repeated reading and data familiarisation. To enhance trustworthiness, the first three transcripts were reviewed in detail by the first author, and 50% of the remaining transcripts were randomly selected for accuracy checking. Disagreements or uncertainties in transcription and data coding were resolved through regular meetings between the RA and the first author. Furthermore, the themes and subthemes were reviewed and confirmed by the research team, who were not involved in the participant recruitment, randomisation, intervention delivery, and outcome assessments. Both positive and negative or ambivalent accounts were retained during the analysis. The results theme table was subsequently translated from Chinese to English by bilingual members of the research team and checked for accuracy and semantic equivalence. Thematic saturation was not used as a stopping criterion because the qualitative component was embedded within a feasibility trial and aimed to explore participant experiences among those allocated to the tgCBFI group.

## 3. Results

**Figure 1:**
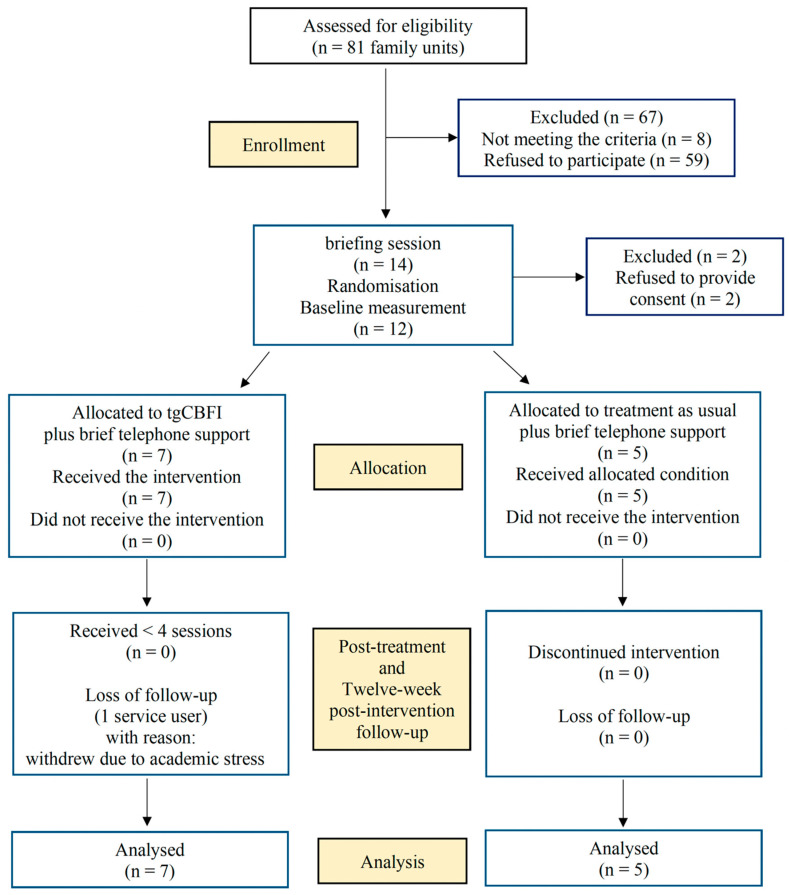
CONSORT flowchart of the tgCBFI study.

Figure 1 shows a CONSORT diagram to illustrate the overview of the study process. In total, 81 family dyads (162 service users and caregivers) were approached for the study, while eight of them were not eligible for the study criteria, 59 of them refused to participate, and two of them refused to provide consent during the briefing session. The most common reasons were lack of interest, time constraints, discomfort with group participation, and unwillingness to involve family members. Since the reasons for refusal were informally collected, the frequency of each reason could not be reported. Eventually, among 73 eligible family dyads, 12 service user–caregiver dyads (24 participants) were successfully recruited from a study site to participate in this study. Seven dyads were allocated to the tgCBFI, and five dyads were allocated to the TAU group. One whole dyad in the tgCBFI group withdrew from the intervention programme after attending four sessions, due to the academic stress of the service user. It was because the intervention programme required the joint participation of service users and caregivers. Except for the withdrawn service user, all the other participants, including the withdrawn caregiver (23 participants, 95.8%), completed all the self-report follow-ups. Both post-treatment and 12-week follow-up outcome data for the only dropped-out service user were imputed using LOCF from his/her baseline data. However, the CSQ-8 score was not imputed because it was measured only post-treatment.

Table 2 and Table 3 show the baseline characteristics of the participants. Service users in the tgCBFI group were older on average than those in the TAU group, and the gender distribution differed between groups. Some clinically relevant baseline imbalances were also observed. TAU group service users had higher baseline PANSS positive symptom scores, whereas caregivers in the tgCBFI group had higher baseline HADS depression scores. Among the service users, most were single (75%), educated up to secondary level (66.7%), unemployed/student (66.7%), with about seven years of average duration of illness, and fully adherent to medications observed by caregivers (83.3%). Among the family caregivers, most were female (75%), married (91.6%), parents (66.7%), and lived in a private flat (75%).

### 3.1. Study Feasibility, Acceptability, and Safety

All participants in the tgCBFI group attended all six online sessions, except one family dyad dropped out after attending the fourth session. The full intervention completion rate was 85.7%, while the completion rate for per-protocol completers (i.e., attending ≥ four sessions) was 100%. However, the target sample size was not achieved, and the recruitment rate was 16.4% (i.e., number of enrolled dyads over number of eligible dyads). The retention rate for the follow-up questionnaires was 95.8%. At post-treatment, the service satisfaction using CSQ-8 rated by both service users (mean score = 27.5, SD = 3.67) and caregivers (mean score = 25.86, SD = 4.71) in the tgCBFI group was positive, achieving ≥80% score of the upper limit of the scale score of 32. No adverse events were reported throughout the study. A finding summary for feasibility outcomes was tabulated for illustration (see Supplementary Table S1). Generally, the overall study feasibility was partially supported, particularly masked by the low recruitment rate (<50%, in the red light of the traffic light system) and additional administrative support for participant recruitment required for study sites, though acceptability among enrolled dyads appears promising (i.e., high intervention adherence and outcome data completion rate categorised in green light).

### 3.2. Service User Outcomes

**Figure 2:**
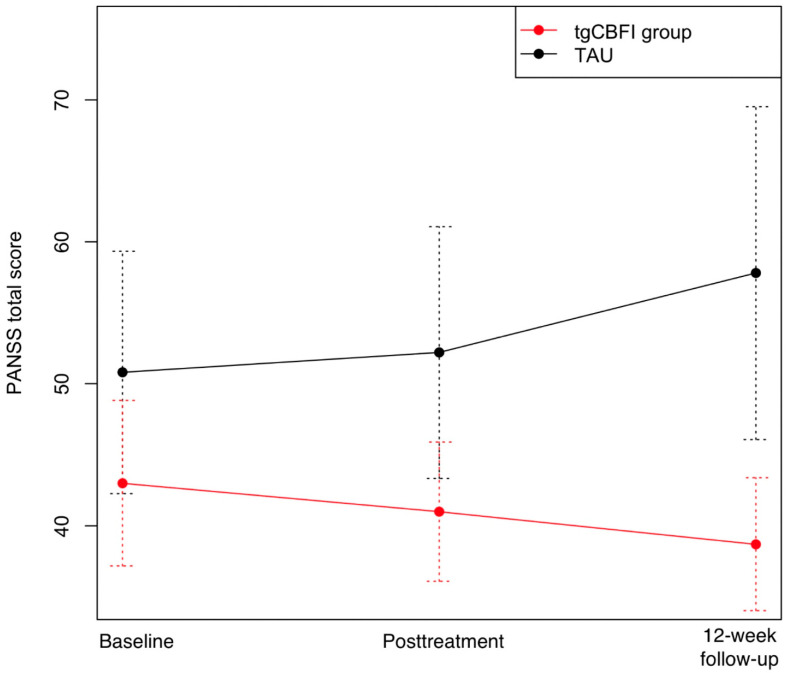
tgCBFI treatment effects on PANSS total score of service users at post-treatment and twelve-week follow-up. Dashed lines and bars indicate the width of the 95% confidence intervals.

Table 4 summarises the tgCBFI treatment effects for service users at post-treatment and twelve-week follow-up. The exploratory analyses suggested that a reduction was observed in the PANSS total score at post-treatment (Hedges’ *g* = −1.51, 95% CI = −2.96, −0.26, *p* = 0.018), and at twelve-week follow-up (Hedges’ *g* = −1.62, 95% CI = −3.10, −0.35, *p* = 0.028). However, despite numerical decreases in the emotional overinvolvement subscale (Hedges’ *g* = −1.28, 95% CI = −2.60, 0.04, *p* = 0.068) at twelve-week follow-up, the expressed emotion total score did not significantly change. Furthermore, the treatment effect size here was exploratory in nature and not considered robust clinical evidence. Figure 2 depicts the PANSS total score change across three time points in a plot to illustrate the longitudinal changes in the tgCBFI treatment effect. A complete case sensitivity analysis was post hoc run to produce similar results to the ITT analysis, with the same exploratory, significant effects in reducing PANSS total and general psychopathology scores at post-treatment and 12-week follow-up (see Supplementary Table S2).

### 3.3. Caregiver Outcomes

Table 5 summarises the tgCBFI treatment effects for family caregivers at post-treatment and twelve-week follow-up. Despite a decrease in small to large effect sizes (Hedges’ *g* ranged from 0.28 to 1.09), neither the levels of anxiety and depression nor the perceived care burden met a significant threshold at post-treatment and twelve-week follow-up.

### 3.4. Themes and Subthemes from Interviews

**Figure 3:**
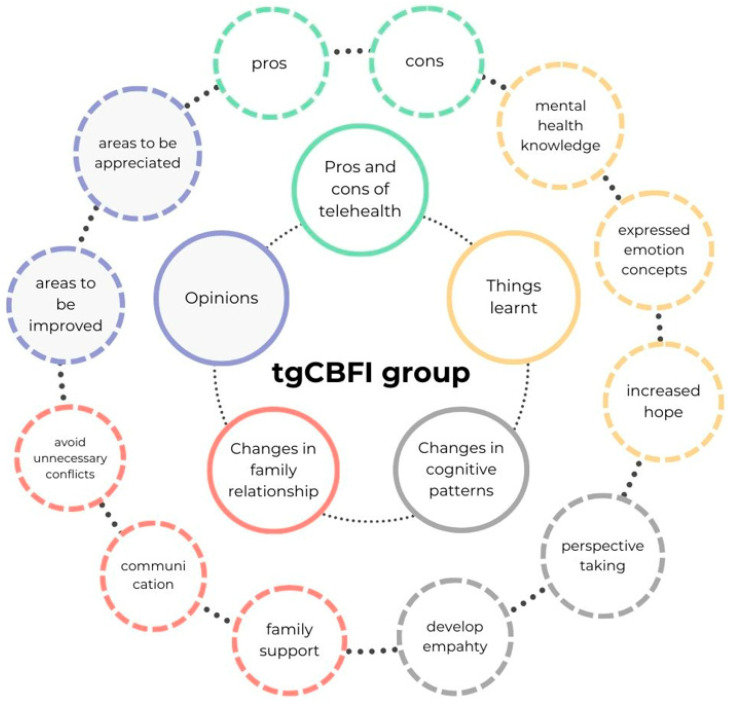
Themes and subthemes generated from the individual interviews for service users and family caregivers in the tgCBFI group. Footnote: solid line circles = themes; dotted line circles = subthemes for their respective themes in the same colour.

Six service users and six caregivers in the tgCBFI group participated in the interviews after completion of the intervention (*n* = 12, 85.7% of the tgCBFI group participants). One service user lost follow-up due to academic stress, and one caregiver did not respond to the invitation. Figure 3 provides an overview of the themes and subthemes generated from the interviews for service users and family caregivers in the tgCBFI group. In addition to themes and subthemes, Supplementary Table S3 summarises the codes and examples of direct quotes from the participants. Five themes, including “Pros and cons of telehealth”, “Things learnt in the tgCBFI group”, “Changes in cognitive patterns”, “Changes in family relationship”, and “Opinions”, and twelve respective subthemes were categorised.

Generally, participants valued the convenience of the tele-group format, the relevance and quality of the content, the session duration, and the interactive nature of the sessions.

*“I have to take care of my children. Having class via Zoom makes my time more flexible. It also saved my travelling time.”*

(caregiver 2)

*“It’s more convenient. You don’t have to wait. You can just log in at the right time without having to prepare for a long time, like taking an hour to get to a place.”*

(service user 15)

*“It’s already well enough. In those six sessions over a few hours, I think it’s okay. For caregivers and patients, it’s a useful course and has a positive impact on them.”*

(caregiver 10)

*“In class, everything was well-organised, with emotions categorised clearly. I hadn’t revised it before, so I was surprised to see how neatly everything was separated into different systems. It provided a systematic way to analyse things, teaching us how to handle situations and think through problems.”*

(service user 10)

They also reported an enhanced awareness of the illness and a deeper understanding of the importance of reducing negative emotional expressions, which fostered hope for the patients’ recovery. They observed positive changes in cognitive patterns and improvements in family dynamics, reflecting meaningful psychosocial impacts of the intervention.

*“The biggest outcome is to view problems from multiple angles and not get stuck in a narrow mindset. Considering issues from different perspectives can definitely inspire me to express more thoughts and ideas.”*

(service user 3)

*“After listening to the instructor, I found a lot of insights, especially in my communication with my husband. It’s really improved a lot; we don’t have as many conflicts as we used to. I’m not as confrontational with him anymore.”*

(caregiver 15)

*The most important thing is that people should mutually respect each other. Of course, we should respect outsiders, but when it comes to our family members, especially children, they also need the same respect and space. They are not your personal property or something you possess; they are independent individuals.”*

(caregiver 9)

However, some concerns were expressed about privacy during online sessions, unstable internet connectivity, and reduced learning atmosphere. Some participants recommended potential improvements, such as adjusting the number of sessions, indicated a preference for individual or face-to-face formats, and requested hard-copy notes.

*“Usually, I might log in about 15 min early to get everything set up. I like to take my time to prepare. I just plan to join right before the start time. But that time, I really couldn’t fix it and ended up being late.”*

(service user 2)

*“If there are other families involved, you might say things you don’t want them to hear. You end up not daring to say too much, feeling like someone might find out, which isn’t ideal. That’s the mindset I have, but others might think it’s not a big deal. I really don’t know.”*

(service user 14)

*“Sharing personal experiences can be challenging, as family members might not feel comfortable discussing their own issues openly. They may prefer individual discussions, where, for example, one person can talk about their case for half an hour while the group focuses separately.”*

(caregiver 14)

*“I think having two weeks of sessions would be fine, like once a week. After each session, we’ve discussed a lot already. Five sessions should be enough, as I see that the content covers the theory well. It seems like the remaining time is mainly for group discussions.”*

(service user 10)

*“The atmosphere of meeting everyone in person and the interaction is much deeper. When there’s a screen in between, it seems like you can’t fully engage with the live experience.”*

(caregiver 9)

*“It might not need to be so strict—either fully online or fully in-person. For the first few classes, it could be beneficial to ease into things since we’re discussing more personal or potentially awkward topics. Once everyone feels more comfortable, transitioning to online sessions or eventually meeting face-to-face in later classes could be a good approach. Or at least for the last session, it would be nice for everyone to meet and see each other. It would be a good opportunity to realise, “Oh, so this is what everyone is like!”*

(service user 7)

*‘It’s not good that we have to print the notes ourselves, because sometimes I forget to do so. If we could have some basic notes provided in advance, that would be much better.’*

(caregiver 14)

## 4. Discussion

This study evaluated the feasibility of tgCBFI for adults with schizophrenia-spectrum disorders and their family caregivers. Although the overall feasibility was only partially supported, mainly because of the low recruitment rate, this is the first RCT examining the feasibility and exploratory effectiveness of tgCBFI (group CBT-based online intervention for family dyads) versus TAU, with brief telephone support, specifically targeting both individuals with schizophrenia-spectrum disorders and their family caregivers. Our findings contribute to the growing empirical findings supporting telehealth-delivered family interventions as a viable approach to overcoming traditional barriers, such as limited accessibility to psychosocial interventions and transportation time to therapy sessions.

Participants, once enrolled in the tgCBFI group, attended all six online sessions, with only one family dyad withdrawing from the intervention programme, resulting in a full intervention completion rate of 85.7% and a 100% completion rate for per-protocol completers (i.e., attending ≥ 4 sessions). Compared with a similar one-group pre-and-post feasibility study on the use of videoconferencing to deliver CBT for individuals with psychosis conducted in Canada [32], the attendance rate of our findings was about 23% higher than that of the previous study, and the attrition rate was similar (i.e., 8.33% in this study versus 10% in that study). The high treatment engagement rate of this study may contribute to the use of the telehealth delivery mode, the interactive nature of sessions, and bi-weekly telephone follow-up, despite potential selection bias for higher treatment-seeking participants being recruited. The videoconferencing format likely enhanced flexible and accessible participation, as demonstrated in previous trials [32,33]. This approach may effectively mitigate common barriers such as geographic constraints and scheduling conflicts that frequently impede traditional in-person family intervention [34], with the resulting increased accessibility potentially facilitating greater participant engagement and retention between sessions [15]. Consistently, qualitative interviews with our study participants also revealed several perceived benefits associated with the use of telehealth in the tgCBFI group. Regarding the recruitment rate of this study, it was far below that of the previous two RCTs using videoconferencing (i.e., 80%) [32,33]. However, in another RCT using mHealth for individuals with serious mental health conditions [35], the recruitment rate was only 33.3%. The possible reason for the low recruitment rate in this study might be the requirement for both service users’ and caregivers’ participation in the intervention group. Multiple strategies to enhance participant recruitment were suggested: a frontline nurse base at the institutions being seconded to the research team of the university, the use of various recruitment channels, and permission for chart review [36]. Strategically highlighting the benefits of the study was also recommended by the participants to enhance participant recruitment.

Nonetheless, technological challenges and motivational barriers inherent to telehealth delivery remain important considerations. In our study, these findings highlight the strengths of tgCBFI in addressing family needs while identifying areas for refinement to optimise engagement and satisfaction. Recent reviews addressing telehealth retention factors echo our qualitative findings and recommend strategies to promote retention [37,38]. Variability in adherence and engagement observed herein and in other telehealth studies highlights the importance of integrating human support components within digital platforms to sustain involvement, maximise intervention adherence, and better clinical outcomes [39,40]. Our findings also suggest that active family involvement not only reinforces therapeutic gains but may be essential in maintaining engagement in telehealth interventions, consistent with the widely recognised significance of family support in schizophrenia care [41,42].

Consistent with prior research demonstrating the effectiveness of CBFI in improving clinical outcomes, our telehealth adaptation showed preliminary results in improving patient symptomatology, both immediately post-treatment and at 12-week follow-up. These results aligned with the previous two pilot studies using videoconferencing-based CBT for individuals with schizophrenia [32,33], highlighting the critical role of family interventions in reducing relapse rates in schizophrenia-spectrum disorders. Future studies may consider examining whether tgCBFI can improve affective symptoms of service users. Although reductions in EE did not reach statistical significance, the tgCBFI group showed a trend toward decreased total EE scores and emotional over-involvement at follow-ups, suggesting potential benefits that may require larger sample sizes or extended follow-up periods to be detected. Similarly, caregiving burden demonstrated favourable but non-significant reductions at follow-ups, indicating that telehealth formats may help mitigate some family burden, albeit with limited effect sizes in this preliminary investigation.

The absence of prominent improvements in caregiver outcomes may be due to several factors. The six-session therapeutic dose and contact may have been insufficient to produce measurable changes in caregiver burden, anxiety, or depressive symptoms. Alternatively, there may be a possible floor effect for the low baseline levels of mood disturbances and perceived care burden for this group of participants. Also, the individual and psychosocial factors related to the caregiving role, such as good family relationships, adequate social support network, positive personality traits, and prior resilient experience [43], may protect from being overburdened in caregiving. However, these variables were not collected and remained uncertain to the caregivers participating in this study. Only the low expressed emotion score rated by service users and the requirement of joint participation in the programme may indirectly reflect that the participating family dyads may be in a neutral or positive family relationship. Additionally, a meta-analysis indicates that higher stress and burnout were found in parental caregivers [44], which accounted for about 70% of our sample. Association or mediation between multiple variables on caregiving experience, perceived care burden, and psychological well-being can be further examined, and specific stress coping for parental caregivers can be focused on.

### 4.1. Limitations

The relatively small sample size of this study may limit the generalisability and statistical power to detect significant effects in expressed emotion, mood symptoms, and caregiver burden. Baseline imbalance is another important limitation. Differences in service users’ age, gender distribution, PANSS positive symptoms, and caregiver depressive symptoms may have influenced subsequent outcome trajectories. Some Hedges’ *g* values were large; however, effect-size estimates from pilot or feasibility trials are often unstable and may overestimate true intervention effects because of small sample sizes and sampling variability. Likewise, the missing data for a service user were imputed by a limited missing data strategy, LOCF, rather than multiple imputation in this feasibility study, and the intervention fidelity was not further maintained by session recording and rating. These may impose a potential therapist allegiance bias on the potentially exaggerated treatment effect and incur reproducibility issues, as the intervention was delivered by a qualified cognitive therapist with a doctoral degree. Also, no correction for multiple testing was applied in the clinical outcomes analysis. These estimates should therefore be interpreted cautiously and were exploratory in an underpowered RCT. The combination of low recruitment, high attendance, and high satisfaction using self-report measures suggests that participants may have represented a more motivated subgroup of service users and caregivers who were comfortable with tele-delivery and family involvement. This may limit generalisability and introduce potential selection bias and self-report response bias, reducing the external validity.

### 4.2. Future Research Directions

The low recruitment rate indicates that participant recruitment requires strategy refinement and collaboration with more study sites before proceeding with a definitive trial. With an adequately large sample size, future definitive trials may consider adding multiple relevant variables, such as caregiving stress and social support, to adjust these covariates in the regression analysis, generating more precise and bias-adjusted estimates of intervention effects. Furthermore, additional treatment content, such as parent-specific modules or more intensive components targeting stress coping and emotional wellbeing, taking into account the current caregiving stress reduction approaches informed by a recent review [45], can be considered. Similarly, refinement of tele-group intervention designs, for example, hybrid, number of sessions, booster sessions, or individual format, needs to be considered to optimise the balance between technological convenience and the critical role of authentic, individualised human support in sustaining participant engagement and enhancing outcomes.

## 5. Conclusions

This small exploratory feasibility study suggests that the use of videoconferencing to deliver group-based cognitive behavioural intervention was partially feasible and demonstrated acceptability among enrolled participant dyads. Preliminary and exploratory findings showed that the tgCBFI may signal potential benefit in reducing clinical symptom severity and encouraging trends toward improving family emotional climate and caregiving burden. A fully powered, rigorous randomised controlled trial is warranted to further examine the intervention programme to elucidate the treatment effectiveness for individuals with schizophrenia-spectrum disorders and their caregivers, before full scalability to the local or international psychiatric services.

## Figures and Tables

**Table 1:** tgCBFI treatment content.

Themes	Content	Objectives
Orientation (session one)	♦ Introduction of the programme ♦ Socialisation of the group participants ♦ Family assessment: genogram illustration ♦ Select one family issue and sharing ♦ Feedback from other dyads of service users and carers ♦ Open discussion ♦ Summary and feedback	♦ Facilitate therapeutic alliance ♦ Brief the expectations and limitations of this programme ♦ Explore family relationships and conflict in the field of mental health ♦ Bridge the communication between service users and their relatives ♦ Foster family mutual support
Psychoeducation (sessions two and three)	♦ Recap the last session’s discussion ♦ Discuss signs and symptoms of schizophrenia-spectrum disorders, stress and perceived care burden of carers ♦ Teach basic concepts of CBT ♦ Automatic thoughts (ATs) and common types of cognitive distortions ♦ Recall a few cases from the last session to demonstrate the learned skills and change the views toward situations ♦ Open up to family context: family interpersonal dynamics ♦ Guided discussion of behavioural interventions for the index family ♦ Compare and contrast the views between sessions one and two on the same family issues ♦ Summary and feedback	♦ Educate on the knowledge of schizophrenia-spectrum disorders ♦ Introduce the CBT model and cognitive distortions ♦ Practise CBT skills and encourage a few participants to reappraise the conflicting situations ♦ Introduce family interpersonal dynamics in the scope of the CBT model ♦ Assist in identifying possible, brief, and practical ways out for the families
Exercise (sessions four and five)	♦ Recap ATs and types of cognitive distortions ♦ Teach basic cognitive and behavioural techniques, such as for and against evidence and role play ♦ Coping skills for residual symptoms and caring tips ♦ Practice and sharing ♦ Summary and feedback ♦ Homework	♦ Do hands-on CBT exercises with personal and/or family case-sharing (invite more participants to raise their concerns) ♦ Teach illness management and caregiving reappraisal ♦ Deliver a piece of CBT homework
Summary (session six)	♦ Recap and consolidation of the CBT model and family approaches ♦ Guided discussion on homework and Q&A of previous sessions ♦ Relapse prevention ♦ Summary and feedback	♦ Summarise the concepts of CBFI and raise awareness of family harmony

**Table 2:** Baseline characteristics of service users (*n* = 12).

	tgCBFI Group	TAU Group	All Participants
**Age**	40.3 (13.6)	23.6 (5.77)	33.3 (13.7)
**Gender**			
male	2 (28.6%)	5 (100.0%)	7 (58.3%)
female	5 (71.4%)	0	5 (41.7%)
**Marital status**			
single	4 (57.1%)	5 (100.0%)	9 (75%)
married	3 (42.9%)	0	3 (25%)
**Education level**			
primary	0	0	0
secondary	3 (42.9%)	5	8 (66.7%)
diploma	2 (28.6%)	0	2 (16.7%)
degree or above	2 (28.6%)	0	2 (16.7%)
**Monthly income**			
unemployment/student	3 (42.9%)	5 (100.0%)	8 (66.7%)
HK$10,000 or below	1 (14.3%)	0	1 (8.33%)
HK$10,001–15,000	1 (14.3%)	0	1 (8.33%)
HK$15,001–25,000	1 (14.3%)	0	1 (8.33%)
HK$25,001–35,000	1 (14.3%)	0	1 (8.33%)
HK$35,001 or above	0	0	0
**Duration of illness** **(in months)**	114 (106.6)	50 (41.9)	87.3 (89.0)
**Number of hospitalisations**	1.86 (2.85)	1.2 (0.84)	1.58 (2.19)
**Number of medication types**	2.28 (1.11)	2.4 (0.89)	2.33 (0.98)
**Medication adherence**			
fully adherent	6 (85.7%)	4 (80.0%)	10 (83.3%)
occasionally missing	1 (14.3%)	1 (20.0%)	2 (16.7%)
most of time missing	0	0	0
not taking at all	0	0	0
**CCLEES**			
total	27.14 (9.118)	22.2 (6.099)	25.08 (8.084)
CC	6.857 (2.854)	7.4 (2.608)	7.083 (2.644)
hostility	9.571 (4.467)	8.2 (3.033)	9 (3.838)
EOI	10.71 (3.904)	6.6 (3.715)	9 (4.221)
**PANSS**			
total	43 (7.853)	50.8 (9.731)	46.25 (9.176)
positive	8.857 (2.035)	12 (2.915)	10.17 (2.823)
negative	11.71 (3.638)	14.2 (3.421)	12.75 (3.621)
general	22.43 (3.78)	24.6 (4.506)	23.33 (4.053)

Footnote: tgCBFI = tele-group cognitive behavioural family intervention; TAU = treatment as usual; CCLEES = Concise Chinese Level of Expressed Emotion Scale, CC = critical comments; EOI = emotional overinvolvement; PANSS = Positive and Negative Syndrome Scale; descriptive data for continuous variables are presented as means and standard deviations, while that for categorical variables are presented as frequencies and percentages.

**Table 3:** Baseline characteristics of family caregivers (*n* = 12).

	tgCBFI Group	TAU Group	All Participants
**Age**	56.43 (16.2)	55.6 (7.89)	56.08 (12.88)
**Gender**			
male	3 (42.86%)	0	3 (25%)
female	4 (57.14%)	5 (100.00%)	9 (75%)
**Marital status**			
single	1 (14.29%)	0	1 (8.33%)
married	6 (85.71%)	5 (100.00%)	11 (91.6%)
**Relationship with service** **users**			
parents	4 (57.14%)	4 (80.0%)	8 (66.7%)
spouse	2 (28.57%)	0	2 (16.7%)
others	1 (14.29%)	1 (20.0%)	2 (16.7%)
**Education level**			
primary	1 (14.29%)	1 (20.0%)	2 (16.7%)
secondary	3 (42.86%)	4 (80.0%)	7 (58.3%)
diploma	0	0	0
degree or above	3 (42.86%)	0	3 (25%)
**Monthly family income**			
HK$10,000 or below	1 (14.29%)	1 (20.0%)	2 (16.7%)
HK$10,001–15,000	3 (42.86%)	2 (40.0%)	5 (41.7%)
HK$15,001–25,000	2 (28.57%)	0	2 (16.7%)
HK$25,001–35,000	1 (14.29%)	0	1 (8.33%)
HK$35,001 or above	0	2 (40.0%)	2 (16.7%)
**Accommodation**			
PHU	2 (28.57%)	0	2 (16.7%)
HOS	1 (14.29%)	0	1 (8.33%)
Private flat	4 (57.14%)	5 (100.00%)	9 (75%)
**Observed medication** **adherence**			
fully adherent	5 (71.43%)	5 (100.00%)	10 (83.3%)
occasionally missing	2 (28.57%)	0	2 (16.7%)
most of time missing	0	0	0
not taking at all	0	0	0
**HADS**			
depression scale	5.587 (2.968)	2.2 (2.49)	4.333 (3.257)
anxiety scale	5.571 (2.699)	2.8 (2.49)	4.417 (2.875)
**FBIS**			
total	15.14 (11.44)	9 (5.788)	12.58 (9.671)

Footnote: tgCBFI = tele-group cognitive behavioural family intervention; TAU = treatment as usual; PHU = public housing unit, HOS = home ownership scheme, HADS = Hospital Anxiety and Depression Scale, FBIS = Family Burden Interview Schedule; descriptive data for continuous variables are presented as means and standard deviations, while that for categorical variables are presented as frequencies and percentages.

**Table 4:** Service user outcomes using change data at post-treatment and twelve-week follow-up.

	tgCBFI Group	TAU Group	Hedges’ g	95% CI	W	*p*-Value
***Post-treatment (n = 12)***						
**CCLEES**						
total	−0.857 (1.952)	−1.4 (4.669)	0.151	(−0.990, 1.309)	16	0.868
CC	−0.143 (1.574)	−1.2 (2.168)	0.532	(−0.612, 1.737)	22	0.499
hostility	0.143 (1.215)	0.2 (1.643)	−0.038	(−1.188, 1.108)	20	0.728
EOI	−0.857 (2.673)	−0.4 (1.517)	−0.185	(−1.346, 0.955)	16	0.866
**PANSS**						
total	−2 (2)	1.4 (2.191)	***−1.510***	***(−2.955, −0.259)***	3	***0.018***
positive	−0.429 (0.787)	0.2 (0.447)	−0.864	(−2.132, 0.302)	10	0.136
negative	−0.571 (1.272)	0.2 (0.447)	−0.694	(−1.928, 0.458)	10.5	0.237
general	−1 (1)	1 (1.732)	***−1.376***	***(−2.779, −0.147)***	3	***0.016***
***12-week follow-up (n = 12)***						
**CCLEES**						
total	−2.143 (3.579)	0.6 (6.986)	−0.485	(−1.683, 0.657)	13.5	0.566
CC	−0.714 (1.976)	−0.8 (4.147)	0.026	(−1.120, 1.175)	17.5	1
hostility	−0.429 (2.149)	−0.2 (2.049)	−0.100	(−1.254, 1.043)	17	1
EOI	−1 (2)	1.6 (1.673)	−1.279	(−2.598, 0.039)	6	0.068
**PANSS**						
total	−4.286 (4.03)	7 (8.916)	***−1.616***	***(−3.096, −0.346)***	3.5	***0.028***
positive	−0.857 (1.215)	−0.4 (1.517)	−0.314	(−1.489, 0.825)	12.5	0.414
negative	−1 (1.528)	1.6 (3.782)	−0.899	(−2.176, 0.269)	8.5	0.156
general	−2.429 (2.370)	5.8 (4.382)	***−2.285***	***(−4.012, −0.874)***	1	***0.009***

Footnote: tgCBFI = tele-group cognitive behavioural family intervention; TAU = treatment as usual; CCLEES = Concise Chinese Level of Expressed Emotion Scale, CC = critical comments; EOI = emotional overinvolvement; PANSS = Positive and Negative Syndrome Scale; descriptive data for continuous variables are presented as means and standard deviations.

**Table 5:** Family outcomes using change data at post-treatment and twelve-week follow-up.

	tgCBFI Group	TAU Group	Hedges’ g	95% CI	W	*p*-Value
***Post-treatment (n = 12)***						
**HADS**						
depression	−2.429 (3.457)	0 (2.915)	−0.690	(−1.922, 0.462)	11	0.325
anxiety	−1.143 (1.464)	1.6 (3.209)	−1.089	(−2.411, 0.100)	7	0.090
**FBIS**	0 (9.487)	3.2 (5.805)	−0.360	(−1.540, 0.780)	12	0.412
***12-week follow-up (n = 12)***						
**HADS**						
depression	−1 (4.082)	0.6 (2.702)	−0.411	(−1.598, 0.729)	16	0.870
anxiety	0 (2.160)	0.8 (3.271)	−0.278	(−1.448, 0.862)	16	0.870
**FBIS**	−3.429 (3.552)	1.8 (10.43)	−0.676	(−1.906, 0.475)	14	0.560

Footnote: tgCBFI = tele-group cognitive behavioural family intervention; TAU = treatment as usual; HADS = Hospital Anxiety and Depression Scale; FBIS = Family Burden Interview Schedule; descriptive data for continuous variables are presented as means and standard deviations.

## Data Availability

The raw data supporting the conclusions of this article will be made available by the authors on request.

## Supplementary Materials

The following supporting information can be downloaded at https://www.mdpi.com/article/10.3390/healthcare14142231/s1, Supplementary Table S1: A finding summary for the feasibility outcomes; Supplementary Table S2: Complete case analysis for service user outcomes using change data; Supplementary Table S3: Themes generated from the individual interviews for six service users and six family caregivers in the tgCBFI group (*n* = 12).
