# Supplementary material for: Tele-Group Cognitive Behavioural Family Intervention for Schizophrenia-Spectrum Disorders and Their Caregivers: A Feasibility Randomised Controlled Trial
Source: Healthcare (Basel). 2026 Jul 22;14(14):2231. doi: 10.3390/healthcare14142231 (PMC13409749; doi:10.3390/healthcare14142231)
Supplement: Supplementary file 1 [file healthcare-14-02231-s001.zip › Supplementary Table S1.pdf]

Supplementary Table S1. *A finding summary for the feasibility outcomes*

Outcomes		Findings
Recruitment rate		16.4%
Per-protocol intervention completion (≥ 4 sessions)		100%
Full intervention completion (6 sessions)		85.7%
Safety		Nil adverse events
Completion of follow-up questionnaires		95.8%
Completion of individual interviews		85.7%
Service satisfaction	for service users	85.9%
	for caregivers	80.8%
Qualitative implementation facilitators		Convenience and willing to share
Qualitative implementation barriers		unstable internet connectivity, compromised learning atmosphere, and privacy concerns

Footnote: Service satisfaction = Mean service satisfaction score over upper limit of the satisfaction scale
