# Supplementary material for: Tele-Group Cognitive Behavioural Family Intervention for Schizophrenia-Spectrum Disorders and Their Caregivers: A Feasibility Randomised Controlled Trial
Source: Healthcare (Basel). 2026 Jul 22;14(14):2231. doi: 10.3390/healthcare14142231 (PMC13409749; doi:10.3390/healthcare14142231)
Supplement: Supplementary file 1 [file healthcare-14-02231-s001.zip › Supplementary Table S2.pdf]

Supplementary Table S2. *Complete case analysis for service user outcomes using change data*

	tgCBFI group	TAU group	Hedges' g	95% CI	W	p-value
Post-treatment (n = 11)						
**CCLEES**						
total	-0.1 (2.098)	-1.4 (4.669)	0.105	(-1.076, 1.300)	13.5	0.853
CC	-0.167 (1.722)	-1.2 (2.168)	0.489	(-0.691, 1.732)	19	0.511
hostility	0.167 (1.329)	0.2 (1.643)	-0.021	(-1.209, 1.165)	17	0.773
EOI	-1 (2.898)	-0.4 (1.517)	-0.230	(-1.486, 1.026)	13.5	0.851
**PANSS**						
total	-2.333 (1.966)	1.4 (2.191)	***-1.650***	***(-3.214, -0.328)***	1.5	***0.015***
positive	-0.5 (0.837)	0.2 (0.447)	-0.926	(-2.262, 0.284)	8	0.131
negative	-0.667 (1.366)	0.2 (0.447)	-0.747	(-2.040, 0.447)	8.5	0.230
general	-1.167 (0.983)	1 (1.732)	***-1.448***	***(-2.941, -0.164)***	1.5	***0.013***
12-week follow-up (n = 11)						
**CCLEES**						
total	-2.500 (3.782)	0.6 (6.986)	-0.521	(-1.769, 0.660)	11	0.52
CC	-0.833 (2.137)	-0.8 (4.147)	-0.010	(-1.197, 1.177)	15	1
hostility	-0.5 (2.345)	-0.2 (2.049)	-0.124	(-1.320, 1.057)	14	0.926
EOI	-1.167 (2.137)	1.6 (1.673)	-1.301	(-2.745, 0.079)	5	0.079
**PANSS**						
total	-5 (3.899)	7 (8.916)	***-1.658***	***(-3.226, -0.335)***	2.5	***0.028***
positive	-1 (1.265)	-0.4 (1.517)	-0.397	(-1.626, 0.781)	10	0.369
negative	-1.167 (1.602)	1.6 (3.782)	-0.907	(-2.238, 0.302)	7	0.157
general	-2.833 (2.317)	5.8 (4.382)	***-2.326***	***(-4.167, -0.852)***	0.5	***0.010***

Footnote: tgCBFI = tele-group cognitive behavioural family intervention; TAU = Treatment as usual; CCLEES = Concise Chinese Level of Expressed Emotion Scale, CC = Critical comments; EOI = emotional overinvolvement; PANSS = Positive and Negative Syndrome Scale; Descriptive data for continuous variables are presented as means and standard deviations
