# Supplementary material for: Tele-Group Cognitive Behavioural Family Intervention for Schizophrenia-Spectrum Disorders and Their Caregivers: A Feasibility Randomised Controlled Trial
Source: Healthcare (Basel). 2026 Jul 22;14(14):2231. doi: 10.3390/healthcare14142231 (PMC13409749; doi:10.3390/healthcare14142231)
Supplement: Supplementary file 1 [file healthcare-14-02231-s001.zip › Supplementary Table S3.pdf]

**Supplementary Table S3.** *Themes generated from the individual interviews for six service users and six family caregivers in the tgCBFI group (n = 12)*

| Theme | Subtheme | Theme description | code | Example of direct quotes |
|---|---|---|---|---|
| **1. Pros and cons of telehealth** | pros | benefits of receiving online psychosocial intervention | convenience | It is more convenient because you don't have to attend the class in person. It saves a lot of time. (10F, p. 1) |
| | | | | I have to take care of my children. Having class via Zoom makes my time more flexible. It also saved my travelling time. (2F, p. 1) |
| | | | | It's more convenient. You don't have to wait. You can just log in at the right time without having to prepare for a long time, like taking an hour to get to a place. (15S, p. 1) |
| | | | willing to share | When you meet someone online, it feels like you can communicate more directly. You can address the issues at hand without beating around the bush or getting tongue-tied. Plus, if you're nervous about saying something wrong, it's a bit easier in a virtual setting. (15S, p. 8) |
| | | | | When I get home, I can share in a more comfortable mode. Plus, seeing other participants face-to-face can sometimes create a bit of pressure, but with a screen in between, it feels more comfortable. (7S, p. 1) |
| | cons | disadvantages of receiving online psychosocial intervention | unstable internet connectivity | When the signal is poor, the conversation can get interrupted. So, it doesn't feel as deep or meaningful as it could be. (9F, p. 13) |
| | | | | Usually, I might log in about 15 minutes early to get everything set up. I like to take my time to prepare. I just plan to join right before the start time. But that time, I really couldn't fix it and ended up being late. (2S, p. 10) |
| | | | compromised learning atmosphere | The atmosphere of meeting everyone in person and the interaction is much deeper. When there's a screen in between, it seems like you can't fully engage with the live experience. (9F, p. 12) |
| | | | | Of course, being there in person is the best. The atmosphere would be amazing. The reliance on online formats is really just due to the circumstances. Sometimes, it's necessary to adapt to the availability of participants and their schedules. (3F, p. 9) |
| | | | privacy concerns | Maybe it's just that the few participants are… um… well-behaved or cooperative, but there could be some who are secretly recording or taking notes. That might be a concern. I mean, I don't mind sharing what I say, but there is that concern and risk involved. (7S, p. 1) |
| | | | | If there are other families involved, you might say things you don't want them to hear. You end up not daring to say too much, feeling like someone might find out, which isn't ideal. That's the mindset I have, but others might think it's not a big deal. I really don't know. (14S, p. 1) |
| **2. Things learnt in the tgCBFI group** | mental health knowledge | gain more knowledge about mental illness and psychotropic drugs | better understanding of mental illness | I've learned that there are actually many of these kinds of conditions, and it often comes down to a lack of something, like dopamine or whatever it is. (2F, p. 2) |
| | | | | I just observed that when someone got sick, it took me a while to realise it was a mental illness, and that's when I learned they were seeing a doctor. Throughout that process, I found out that they could get better. However, for certain reasons, they might stop taking their medication, and then they could relapse. That's how I learned about these things. I'll understand that you have to learn to live with it. (10F, p. 2) |

| | | | | |
|---|---|---|---|---|
| | | | importance of taking medications | We originally didn't understand that taking medication was important, thinking that if there were no issues, there was no need for it. We didn't realise that these conditions are related to a deficiency in the brain. We assumed that if there were no memory problems or unhappiness, everything was stable, and we wouldn't need medication. We often hoped to stop taking it. Now we understand that this condition involves a lack of something in the brain, and that means you have to take that medication; you can't skip it. (2F, p. 1) |
| | | | | To minimise the chances of a relapse, make sure they take their medication regularly. (10F, p. 7) |
| | | | reflection on medication adherence | I noticed that after she stopped her medication for about a month, her mental state and overall well-being declined. It started with a low dose of three milligrams, but now it has increased to six milligrams. These are things the teacher mentioned. It's important to understand that this medication isn't like taking something for a cold, where you stop once you feel better. We need to grasp these concepts and learn from them. I've realised my own mistakes in this process. (3F, p.17) |
| | | | | My wife has the mindset that it's best not to take medication. Initially, she thought it was necessary, but she feels that once you recover from an illness, like a cold, you wouldn't keep taking cold medicine every day. She thought she could stop and gradually reduce the dosage. However, her experience showed that it can quickly lead to a relapse. She realised from the literature that not everyone can manage that way. Many people need to rely on medication permanently to help them cope. (10F, p. 2) |
| | expressed emotion concepts | understand the impact of family atmosphere on the mental wellbeing of service users | family arguments and poor communication would negatively influence the service users | If I were the one experiencing a mental health crisis, and someone else kept bringing up the triggers for my condition, it would likely increase the chances of a relapse. Instead, if they didn't mention those triggers and focused on positive solutions or activities, it could really help. Redirecting my attention to other things, like engaging in fun activities or doing chores, would help shift my mindset away from the stressor. Keeping the mind occupied with different, less stressful tasks can help resolve the issue. Constantly looping back to the same problem only reinforces it, making it harder to cope. (10S, p. 7) |
| | | | | Family members with mental health issues may be more sensitive to loud noises, questioning, or unfriendly conversations. These situations can easily trigger their anxiety or discomfort, making it important to approach them with care and understanding. (7S, p. 4) |
| | increased hope for recovery | believe service users could live independently | service users could take care of themselves | Having a mental illness doesn't mean it's a dead end. She can achieve a lot when she is stable. There's really no difference from someone without a mental health issue. If you didn't tell me she had a condition, I wouldn't even notice. She can maintain good mental health and continue to pursue whatever she wants to try. (3F, p.14) |
| | | | | She's quite stable now and doesn't really need my help. Instead, she often takes care of us by making desserts and cooking. It feels nice that she looks after us, and it doesn't feel like we have to take care of her anymore. (2F, p. 9) |
| **3. Changes in cognitive patterns** | perspective taking | learn to think from another perspective | positive thinking | I think the key to the analysis is how to view a situation using a different perspective to look at the matter. It doesn't always have to be negative. You can see a more positive side to it from another angle. (10F, p. 2) |

| | | | | |
|---|---|---|---|---|
| | | | | It turns out that when it comes to thinking things through or solving problems, it's important not to get stuck in a narrow mindset. Being able to consider different aspects, both the positive and the negative, can really help in finding solutions. (10S, p. 2) |
| | | | | The biggest outcome is to view problems from multiple angles and not get stuck in a narrow mindset. Considering issues from different perspectives can definitely inspire me to express more thoughts and ideas. (3S, p. 1-2) |
| | | | | When the teacher provided us with different examples, I realised that I could be subtly influenced by the cognitive behavioural patterns. For instance, even if I initially disagree or feel a certain way, I might sense that you're upset or that there are aspects I need to understand. As a result, I might choose to adjust my response, such as deciding to speak out later, expressing my thoughts at a more appropriate time, or finding another way to communicate. (7S, p. 3) |
| | | | emotion regulation | By thinking from a different perspective, I can calm myself down. I consider the situation in a more positive light, like realising that if you express what you don't like, it's actually helpful. This way of thinking makes me feel happier. (10S, p. 5) |
| | | | | If others see me as having a problem, I naturally feel a bit hurt inside. But I try to understand their perspective, even if I can't respond immediately. I think about how they might feel first, which helps me take a moment to calm down and prepare myself before reacting. (7S, p. 3) |
| | develop empathy | having a new perspective and developing empathy | being more empathetic | In the past, I just accepted things without much understanding or agreement. I would simply go along with whatever she said. But after attending these sessions, I understand why she thinks that way and why those thoughts persist. (14F, p. 4) |
| | | | | To be able to forgive others, you need to learn to forgive yourself and understand their perspective. In the past, I would just focus on my own thoughts without considering how the other person might feel. (15F, p. 1) |
| **4. Changes in family relationship** | family support | recognise the importance of family support | encouragement and affirmation from family caregivers | Sometimes, it's really important to provide more encouragement and affirmation. For example, reminding my daughter that she's faced similar situations before and ultimately resolved them can help. If she only focuses on her current struggles, she might forget her past successes. Those experiences can build her confidence to face challenges. So, giving her more affirmation and encouragement is crucial. (9F, p. 12) |
| | | | | She gives me confidence, reminding me not to be afraid. She says that dealing with this illness takes time, and that our family is here to help me overcome it together. Even though my mom doesn't say much, I can feel her support by my side. (3S, p. 12) |
| | communication | improve communication within a family | communicate more with family members | Communication has become more meaningful, as I'm now making an effort to listen more attentively. In the past, we were too familiar with each other and didn't have deep conversations. Now, we've started to have more heartfelt discussions. (3S, p. 6) |
| | | | | Now, we communicate more directly and don't hold things back. Since it's just the two of us at home, there used to be misunderstandings, and after work, we were both busy and didn't really want to talk. But now, if something comes up, even if it's not immediately addressed, we might message each other on WhatsApp, saying, "Hey, let's talk about this tonight," which helps us remember to discuss it. Even if we can't resolve everything quickly, we can tackle things one by one, which makes our interactions more positive. (7S, p. 3) |

| | | | | |
|---|---|---|---|---|
| | | | | We have more communication than before, and the relationship with my family has become more open. During the group sessions, we were asked to think of certain cases involving the people we interact with most. We shared any conflicts or issues we had, which meant that others, like my husband, could hear about my concerns. Normally, I wouldn't express these feelings, but now that they're out in the open, we can see each other's thoughts and feelings. It helps us understand what each other likes or dislikes, which is really valuable. (10S, p. 4-5) |
| | | | | After completing these sessions, the main change seems to be that I've become more attentive to what he says. I've learned to listen more actively. (10F, p. 4) |
| | | | mutual respect and tolerance | The most important thing is that people should mutually respect each other. Of course, we should respect outsiders, but when it comes to our family members, especially children, they also need the same respect and space. They are not your personal property or something you possess; they are independent individuals. (9F, p. 2) |
| | | | | I tend to be a more structural person, probably because I studied the humanities. My family members, on the other hand, are involved in sports, where you just think of something and go for it. Also, our work styles are different; I work in an office where things aren't urgent, so I approach tasks step by step. However, she works in frontline management, where someone might get injured and there's no time to wait. So, our personalities might differ because of these factors. (7S, p. 6) |
| | avoid unnecessary conflicts | avoid or reduce the family interpersonal conflicts | avoid conflicts | When I encounter problems now, I've learned how to deal with them and avoid them. I just step outside, go somewhere else, and leave the situation. (15S, p. 4) |
| | | | | If someone wants to criticise you, it's best not to focus on whether it makes sense or not. Instead, try to handle it gently and diplomatically. Using a more subtle approach can often lead to a better outcome. (7S, p. 5) |
| | | | reduced episodes of conflicts | So, there are fewer conflicts overall, but some still arise. He might notice things when I do something that doesn't align with his expectations, and he can get angry. Now, he might realise that he should understand my perspective sometimes. It's not like taking this course has solved everything, but we know we can talk about issues instead of just sitting in silence when upset. We're better at expressing ourselves now. (10S, p. 5) |
| | | | | After listening to the instructor, I found a lot of insights, especially in my communication with my husband. It's really improved a lot; we don't have as many conflicts as we used to. I'm not as confrontational with him anymore. (15F, p. 2) |
| **5. Opinions** | areas to be appreciated | worth appreciating the components of the intervention content and arrangements | modest length of each session | Yes, that's right. If the duration of the class was too long, it's understandable to feel overwhelmed. But just like studying, you can handle it in shorter bursts, like about 90 minutes or so, and then take a break after about half an hour. (3F, p. 10) |
| | | | | That's right, about half an hour is just right. Not too long. Two hours would be too much, but half an hour is a good balance. (14S, p. 10) |
| | | | Q&A sessions regarding mental health topics | During class, there's more time to chat with the instructor. It's not just a few minutes; I consider myself lucky that he always responds with three words or more. However, since doctors can be busy, sometimes I have to go through the community health nurse to get in touch. That process isn't very effective. If I have a question, I have to call the nurse, who then contacts the doctor, and the doctor replies to her before she gets back to me. But in class, even though I only have that specific time each week, I'm confident I can get immediate answers. (7S, p. 2) |

| | | | |
|---|---|---|---|
| | | | Our teacher is very clear about the medications. During class, for example, if someone or I ask about the drugs, he can answer our questions. (14S, p. 10) |
| | | | The Q&A segment is really special. Having the opportunity to ask questions in every class is great. Sometimes there are things you don't know, and being able to ask is very helpful. I think that's excellent! (2S, p. 4) |
| | | opinions and daily examples shared by group members | You can hear the opinions of people from other groups, which provides different perspectives. It helps you realise that you're not the only one experiencing certain emotional issues, and it's valuable to see how others approach them. (10S, p. 1) |
| | | | There are some real-life examples and people's sharing that make it more relatable. This helps you remember things much better and makes the lessons more impactful. (15S, p. 2) |
| | | concise and organised teaching content | It's already well enough. In those six sessions over a few hours, I think it's okay. For caregivers and patients, it's a useful course and has a positive impact on them. (10F, p. 8) |
| | | | In class, everything was well-organised, with emotions categorised clearly. I hadn't revised it before, so I was surprised to see how neatly everything was separated into different systems. It provided a systematic way to analyse things, teaching us how to handle situations and think through problems. (10S, p. 3) |
| | | | The content is quite good. He provided us with notes and emailed them to us, so even after the class, if we forget something, we can refer back to them and review as needed. (14S, p. 2) |
| | | case studies and analyses | I really liked the case analysis and the explanation of the cognitive framework. I enjoy reading more text-based material because it helps clarify things. When the explanations are detailed, it makes it easier to understand. (15S, p. 2) |
| | | | He provided many examples for us to analyse, which were both in-depth and easy to understand. We found it very helpful. (3S, p. 2) |
| | | | The discussion involves analysing the entire situation and organising the issues, then reflecting on how to view things differently. The instructor also shares examples of how to approach each case study, showing us how to apply those ideas. It's about shifting our perspective to be less negative and finding more positive ways to tackle the problems. (10F, p. 3) |
| | areas to be improved | recommendations to improve the intervention design | individual sessions | Sharing personal experiences can be challenging, as family members might not feel comfortable discussing their own issues openly. They may prefer individual discussions, where, for example, one person can talk about their case for half an hour while the group focuses separately. (14F, p. 3) |
| | | | | For example, if someone is currently experiencing anxiety and depression, the few hours spent in a Zoom class—let's say ten hours total—might not feel like a lot of learning. It's not like one-on-one interaction, so it would be beneficial to focus on different cases and have deeper discussions. (9F, p. 13) |
| | | | number of sessions | I can't think of specific questions because there are some topics I'm not very familiar with, so I don't know what to ask. After listening, I realise, "Oh, so that's how it is." I'd like to have at least two more sessions, or maybe even four if they're longer. Even just two more would be great! (2S, p. 9) |
| | | | | I think having two weeks of sessions would be fine, like once a week. After each session, we've discussed a lot already. Five sessions should be enough, as I see that the |

| | | |
|---|---|---|
| | | content covers the theory well. It seems like the remaining time is mainly for group discussions. (10S, p. 7) |
| | hybrid format by adding a face-to-face session | It might not need to be so strict—either fully online or fully in-person. For the first few classes, it could be beneficial to ease into things since we're discussing more personal or potentially awkward topics. Once everyone feels more comfortable, transitioning to online sessions or eventually meeting face-to-face in later classes could be a good approach. Or at least for the last session, it would be nice for everyone to meet and see each other. It would be a good opportunity to realise, "Oh, so this is what everyone is like!" (7S, p. 3 & 9) |
| | | It would be nice to add a session where we can all sit together, even if it's still online. Maybe we could have a part where we can see each other, like a more interactive session for us to meet one another. I think that would be really good for building connections. (2S, p. 5) |
| | level of difficulty | Some of the examples were a bit too difficult for me to grasp initially. The six examples were quite challenging to understand. However, once the instructor analysed them for us, it became easier to comprehend. I hope future classes could include simpler examples that help clarify the underlying concepts more effectively. (3S, p. 4) |
| | | I feel that just one hour a week isn't enough for what we're learning. I hope that patients can receive more in-depth support and services under the guidance of the instructors. (9F, p. 13) |
| | state the benefits during participant recruitment | They need to understand the benefits of participating. People can be quite realistic, and they often ask what the advantages are. If you can clearly outline the benefits, hitting the right points, they'll be more inclined to want to join in. Alternatively, you might need to emphasise whether there's a vacancy for them to join. (10F, p. 8) |
| | | It's possible that some people might feel a certain way about being referred because they have a problem. For me, personally, I don't mind, but I think it would be better to frame it differently. Instead of saying, "We're recruiting you because of this issue," we could say something like, "We want to help you make breakthroughs or improvements in this area." This way, it feels more positive and encouraging, rather than just focusing on the problem. (7S, p. 8) |
| | hard copy notes | It's not good that we have to print the notes ourselves, because sometimes I forget to do so. If we could have some basic notes provided in advance, that would be much better. (14F, p. 1) |
| | | I hope we can have some printed notes to help us delve deeper into the material. In class, time goes by so quickly that we remember things in the moment, but when we get back, we often want to summarise what we learned. Having physical notes would allow us to reinforce our memory and understand how to apply what we've learned in our daily lives. Since these topics are things, we rarely encounter, combining printed materials with practice could help us better integrate theory and practice, making it easier to implement what we've learned. (9F, p. 1) |

Footnote: In the brackets, S = service user; F = family caregivers, p. = page number of the interview transcripts
